# Tigliane and daphnane diterpenoids from Thymelaeaceae family: chemistry, biological activity, and potential in drug discovery

**DOI:** 10.1007/s11418-023-01713-x

**Published:** 2023-06-09

**Authors:** Kouharu Otsuki, Wei Li

**Affiliations:** Faculty of Pharmaceutical Sciences, Toho University, Miyama 2-2-1, Funabashi, Chiba 274-8510 Japan

**Keywords:** Tigliane, Daphnane, Diterpenoid, Thymelaeaceae

## Abstract

Tigliane and daphnane diterpenoids are characteristically distributed in plants of the Thymelaeaceae family as well as the Euphorbiaceae family and are structurally diverse due to the presence of polyoxygenated functionalities in the polycyclic skeleton. These diterpenoids are known as toxic components, while they have been shown to exhibit a wide variety of biological activities, such as anti-cancer, anti-HIV, and analgesic activity, and are attracting attention in the field of natural product drug discovery. This review focuses on naturally occurring tigliane and daphnane diterpenoids from plants of the Thymelaeaceae family and provides an overview of their chemical structure, distribution, isolation, structure determination, chemical synthesis, and biological activities, with a prime focus on the recent findings.

## Introduction

Thymelaeaceae family is distributed throughout the world except in the arctic zones, with 53 genera and more than 800 species [1, 2]. Most of them are trees or shrubs, rarely perennial or annual. The plants of this family are not only popular as ornamental trees but also have a wide range of uses including incense, paper-making material, and traditional medicine. For example, agarwood, a non-timber resinous wood obtained mainly from the genera *Aquilaria* and *Gyrinops*, has a long history as a high-grade incense and has played an important role in the traditional medicine of Asian Nation [3]. Bast fibers collected from *Edgeworthia chrysantha* and *Wikstroemia sikokiana* have long been used to make high-quality paper and banknotes in Japan [4]. Some of them, such as *Daphne genkwa* and *Stellera chamaejasme*, have a long history as medicinal plants used in traditional Chinese medicine [5].

Tigliane and daphnane diterpenoids, which are characteristically distributed in plants of the Thymelaeaceae family as well as the Euphorbiaceae family, are structurally diverse due to the presence of polyoxygenated functionalities in the polycyclic skeleton. These diterpenoids, such as phorbol esters of tigliane and daphnetoxin of daphnane, are known as toxic components, while they have been shown to exhibit a wide variety of biological activities and are attracting attention in the field of natural product drug discovery.

**Fig. 1:**
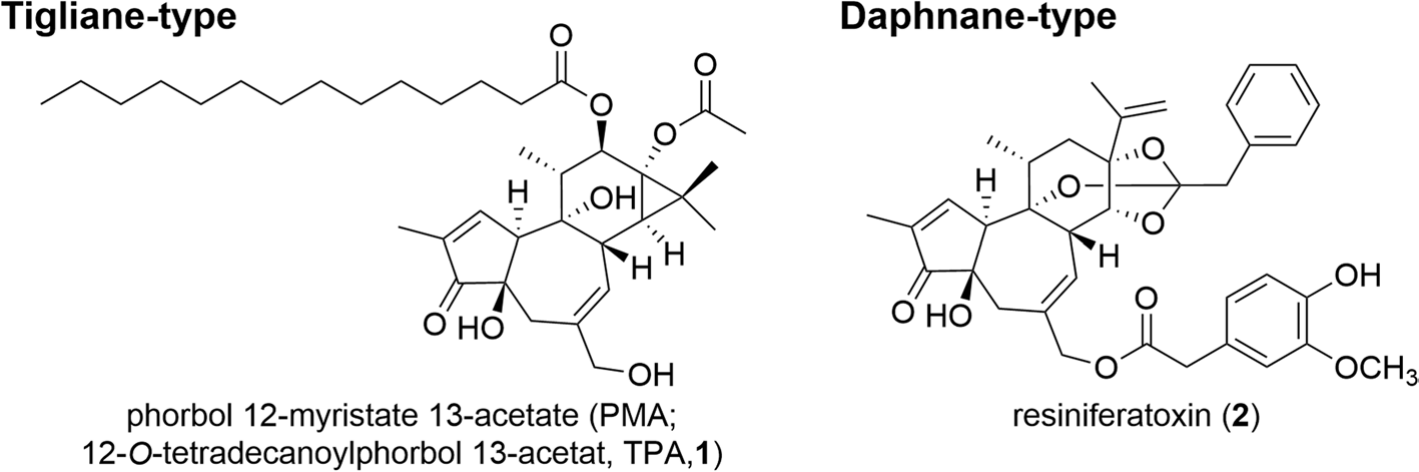
Representative phorbol ester of tiglianes and daphnanes

12-*O*-Tetradecanoylphorbol 13-acetate (TPA; phorbol 12-myristate 13-acetate, PMA, **1**), a tigliane diterpenoid also known as phorbol ester, is a potent tumor promoter that exerts its effects through the activation of protein kinase C (PKC) (Fig. 1) [6]. It has been utilized as a reagent in pharmacological studies to activate downstream signaling pathways as a PKC agonist. Resiniferatoxin (**2**), a daphnane diterpenoid, is an analog of capsaicin that shows potent analgesic effects by stimulating transient receptor potential vanilloid 1 (TRPV1) [7–9]. It is expected to be developed as a non-addictive analgesic with reduced side effects in comparison with morphine, since it does not act on opioid receptors. Currently, phase II and III clinical trials are underway to evaluate the efficacy of **2** for the management of cancer pain and knee osteoarthritis-related pain [10].

Attractive tigliane and daphnane diterpenoids have previously been described in reviews by Liao et al. in 2009 and Wang et al. in 2015 [11, 12]. However, since the publication of these reviews, further advancements have been made in the research of naturally occurring tigliane and daphnane diterpenoids. Therefore, this review focuses on naturally occurring tigliane and daphnane diterpenoids from plants of the Thymelaeaceae family and provides an overview of their chemical structure, distribution, isolation, structure determination, chemical synthesis, and biological activities, with a prime focus on the recent findings.

## Chemical structure

**Fig. 2:**
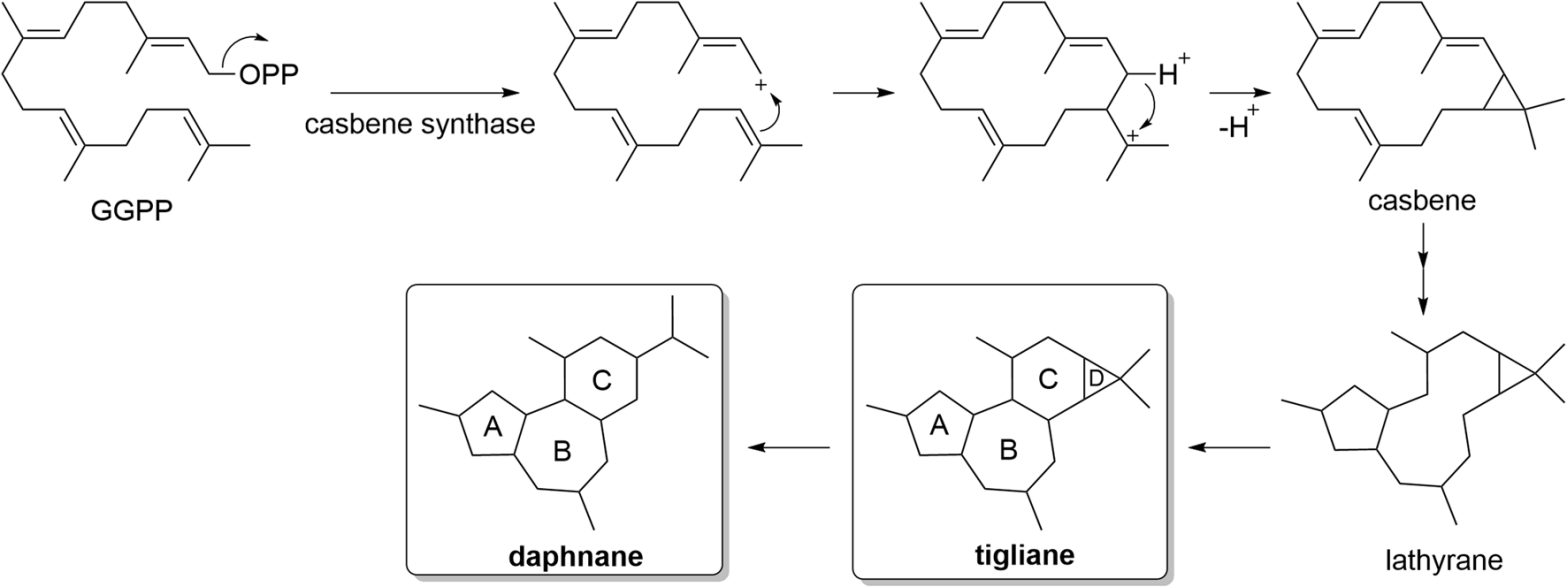
Biosynthesis of tigliane and daphnane diterpenoids

Tigliane and daphnane diterpenoids are considered to be biosynthesized from casbene. While the exact mechanisms have not been fully elucidated, it is thought that the reaction catalyzed by casbene synthase from geranylgeranyl diphosphate (GGPP) generates casbene, which undergoes ring-closing reactions to form lathyrane, leading to tigliane. Then, the cyclopropane ring of tigliane opens to form an isopropyl group to form daphnane (Fig. 2). These skeletons are further modified by various oxygenation and esterification reactions, giving rise to tigliane and daphnane diterpenoids with a diverse array of chemical structures [13, 14].

**Fig. 3:**
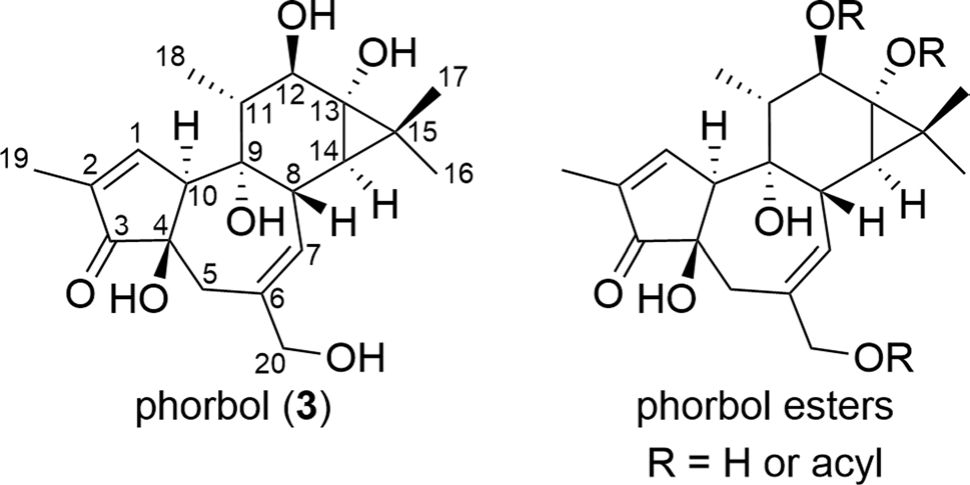
Structure of phorbol and phorbol esters

**Fig. 4:**
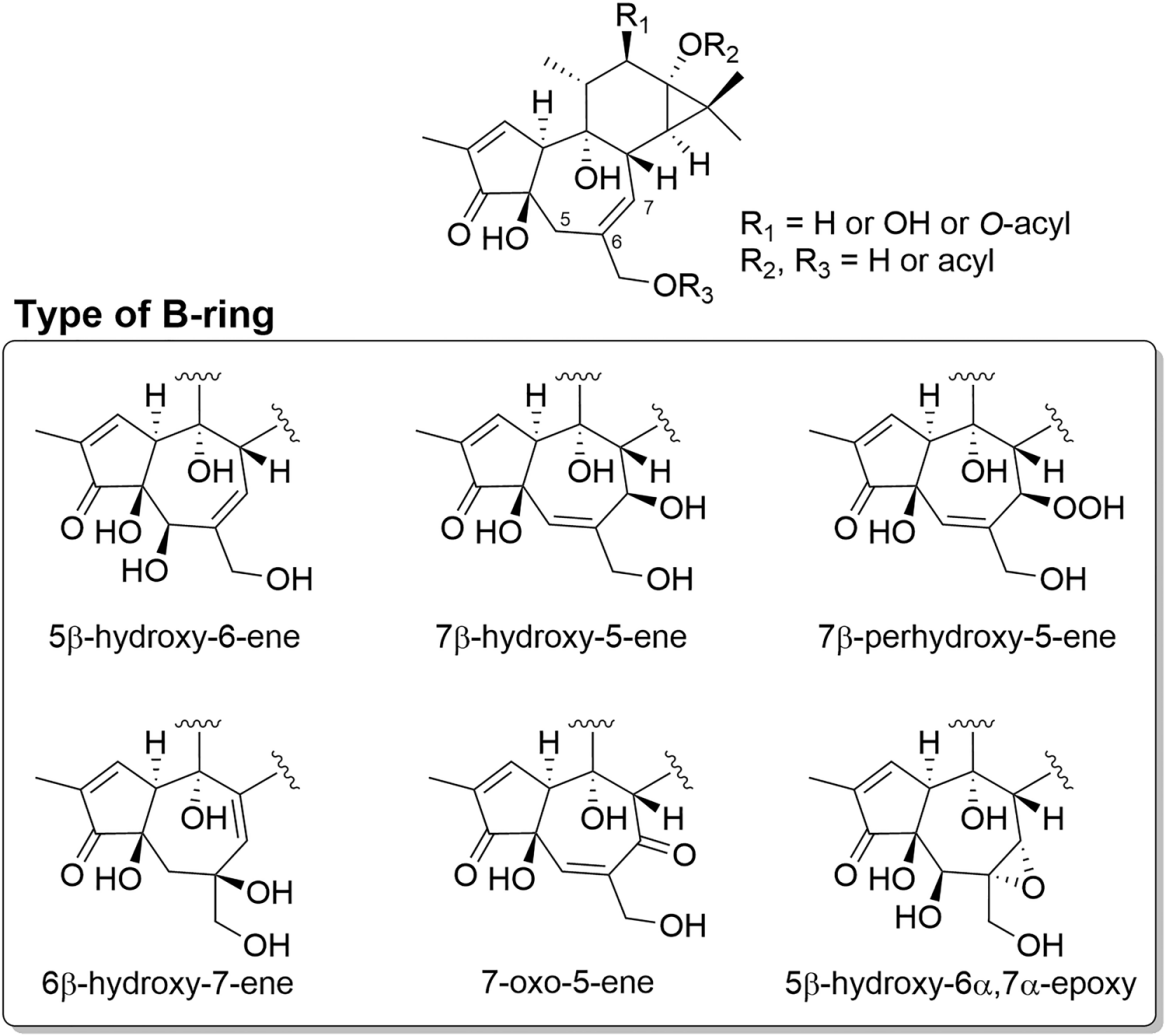
Structure of tigliane diterpenoids isolated from plants of the Thymelaeaceae family

Tigliane diterpenoids possess a 5/7/6/3 (A/B/C/D) fused tetracyclic structure, wherein the D-ring forms a *gem*-dimethylcyclopropane ring. In all of tigliane isolated from plants of the Thymelaeaceae family, the A/B and B/C rings are *trans*-fused, while the C/D ring is *cis*-fused. Tigliane with an α,β-unsaturated ketone structure in the A-ring, a double bond between C-6 and C-7, a primary hydroxy group at C-20, a secondary hydroxy group at C-12, and tertiary hydroxy groups at C-4, C-9, and C-13 are known as phorbol (**3**). The group of compounds in which the hydroxy group at C-12, C-13, or C-20 of phorbol is esterified is typically referred to as phorbol esters, representing the most canonical class of tiglianes (Fig. 3). In addition, there is 12-deoxytigliane, which lacks a substituent attached to C-12. Although most of the known tiglianes have been isolated from plants of the Euphorbiaceae family, those isolated from the Thymelaeaceae family are with diverse patterns of oxidative modification of the B-ring, showing the variety of skeletal parts (Fig. 4).

**Fig. 5:**
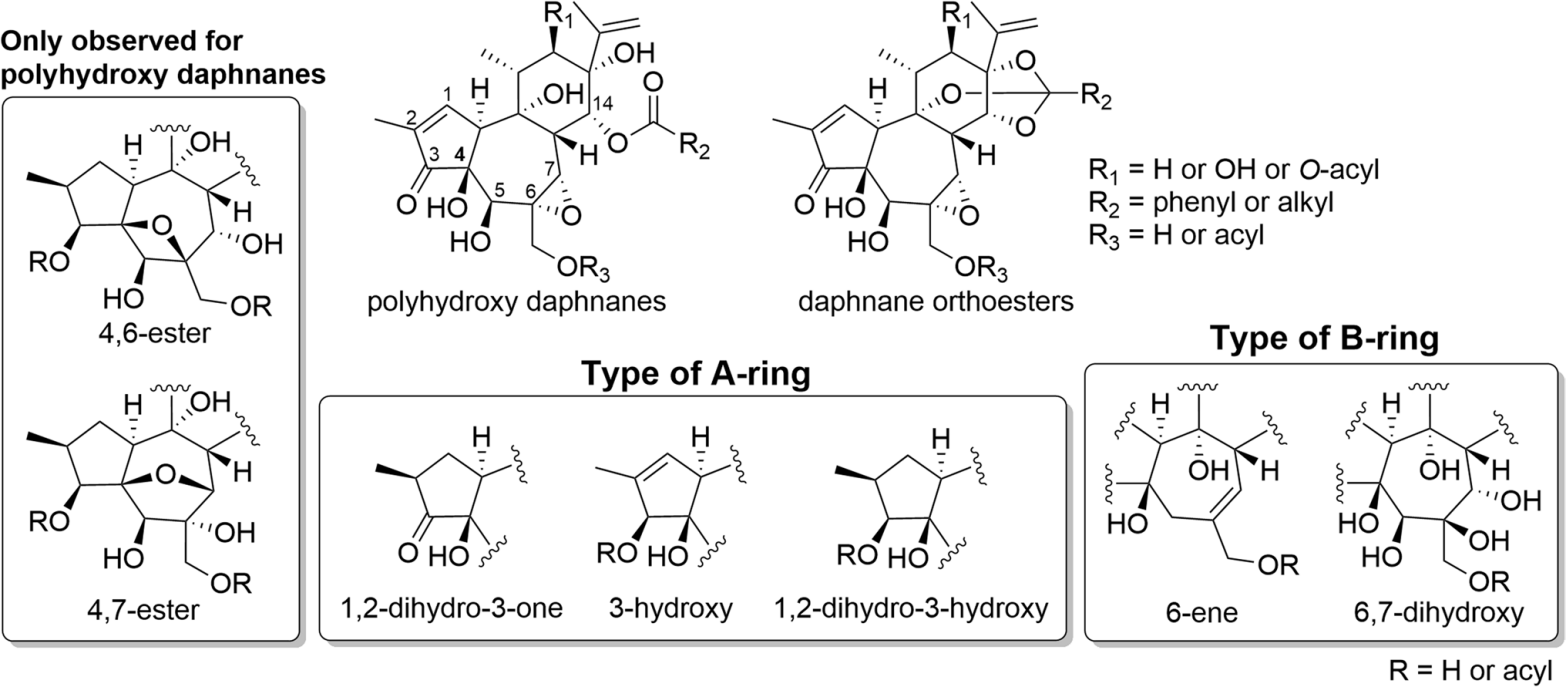
Structure of polyhydroxy daphnanes and daphnane orthoesters isolated from plants of the Thymelaeaceae family

**Fig. 6:**
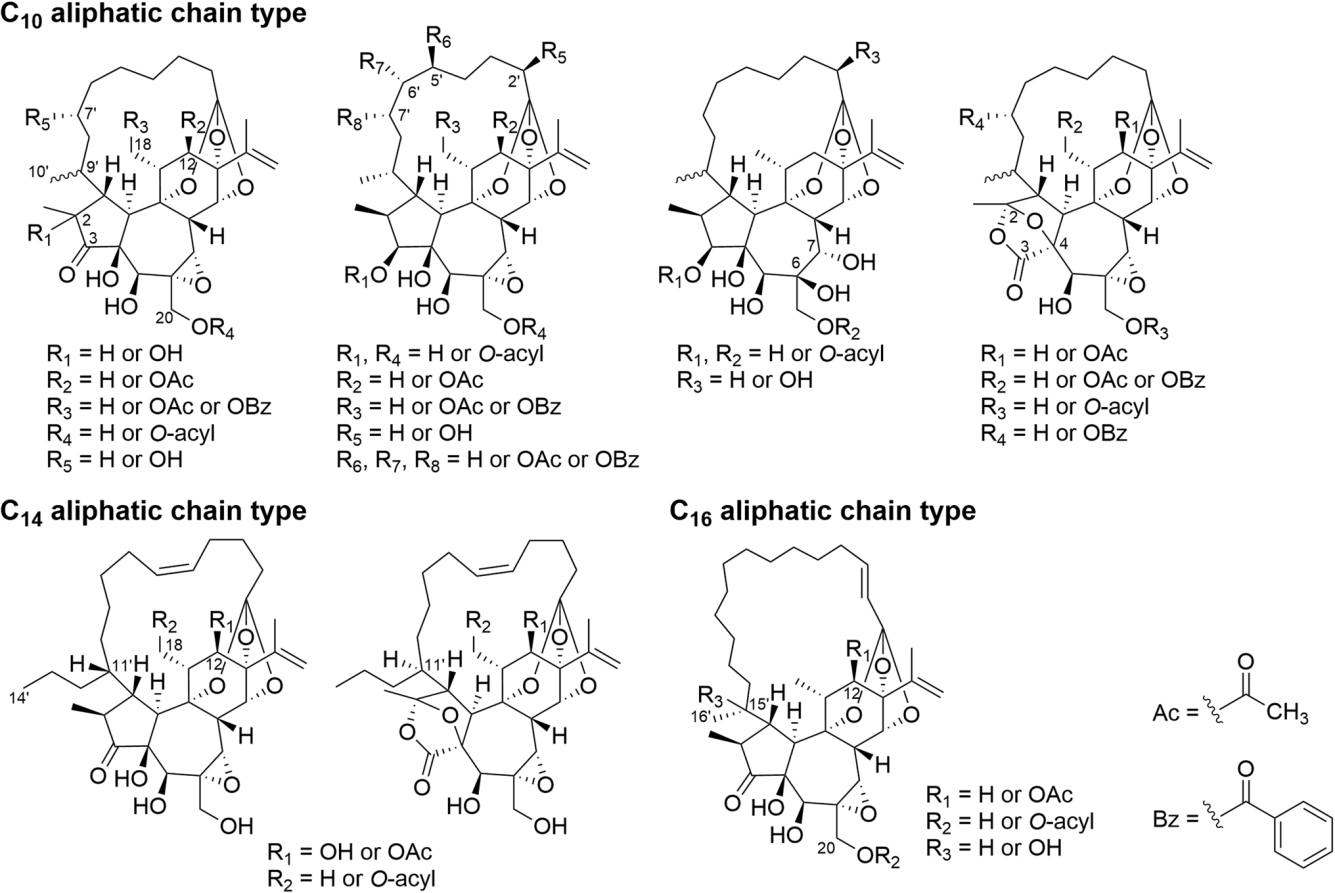
Structure of macrocyclic daphnane orthoesters (MDOs) isolated from plants of the Thymelaeaceae family

On the other hand, the majority of daphnane diterpenoids are isolated from plants of the Thymelaeaceae family rather than the Euphorbiaceae family. Daphnanes possess a 5/7/6 (A/B/C) *trans*-fused tricyclic structure, which is characterized by an isopropenyl group attached to the C-ring. Daphnanes isolated from the Thymelaeaceae family can be classified into three types based on their chemical structural characteristics. The first type is the orthoester daphnane type, which is the most commonly found structure among daphnanes, characterized by an orthoester acylate formed at C-9, C-13, and C-14 of the C-ring (Fig. 5). The second type is the polyhydroxy daphnane type, which does not form orthoester acylate but has a polyhydroxy structure, with most compounds esterified at C-14. The third type is the macrocyclic daphnane orthoester (MDO) type (1α-alkyldaphnane), which has only been isolated from plants of the Thymelaeaceae family. In this type, the aliphatic chain with an orthoester linkage to C-9, C-13, and C-14 is connected to C-1 of the A-ring, forming a macrocyclic ring that spans the skeleton (Fig. 6). The oxidative modification patterns of orthoester daphnanes and polyhydroxy daphnanes types are similar, and both types typically have an α,β-unsaturated ketone in the A-ring, as well as a 6,7-epoxy group in the B-ring. Similar to tiglianes, there are daphnanes with either hydroxy or acyloxy groups attached to the C-12 of the C-ring as well as daphnanes with no substituents attached. Additionally, daphnanes with a 1,2-dihydro, 3-hydroxy (or 3-acyloxy), 1,2-dihydro-3-hydroxy (or 3-acyloxy) structure for the A-ring and 6,7-hydroxy structure for the B-ring have been identified. The polyhydroxy daphnanes are also characterized by the presence of compounds with 4,6-epoxy and 4,7-epoxy structures. The MDOs are divided into three categories based on the length of aliphatic chains spanning the diterpene skeleton: C_10_ aliphatic chain, C_14_ aliphatic chain, and C_16_ aliphatic chain. Among them, the C_10_ aliphatic chain type is the most popular and shows the greatest chemical structural diversity. The C_10_ and C_14_ aliphatic chain types can be further classified into compounds with a five-membered ring structure or a bicyclo ring structure for the A-ring, while only compounds with a cyclopropanone structure for the A-ring have been reported for the C_16_ aliphatic chain type.

## Distribution

Phytochemical investigations on plants of the Thymelaeaceae family have reported the isolation of diterpenoids from 17 genera (Table 1), most from *Daphne*, *Stellera*, *Wikstroemia*, and *Pimelea*. Among them, the plants being most extensively studied and most abundant source of diterpenoids are *Daphne genkwa* and *Stellera chamaejasme*, which have also been used in traditional Chinese medicines. Orthoester daphnanes are the most commonly distributed diterpenoids in plants of the Thymelaeaceae family. However, only tiglianes have been reported from *Aquilaria*, and only MDOs from *Dirca* and *Edgeworthia*. Among MDOs, the distribution of C_14_ and C_16_ aliphatic chain types is very limited, with the C_14_ aliphatic chain type being reported from *Edgeworthia* and the C_16_ aliphatic chain type being reported from *Synaptolepis* [15–18].

**Table 1:** Distribution of tigliane and daphnane diterpenoids in the Thymelaeaceae family

Genus	Tigliane	Daphnane		
		Orthoester	Polyhydroxy	MDO
*Aquilaria*	✓			
*Daphne*	✓	✓	✓	✓
*Daphnopsis*	✓	✓		✓
*Diarthron*		✓	✓	
*Dendrostellera*		✓		
*Stelleropsis*	✓	✓	✓	
*Dirca*				✓
*Edgeworthia*				✓
*Gnidia*		✓		✓
*Lasiosiphon*		✓		
*Peddiea*		✓		
*Phaleria*		✓		✓
*Pimelea*	✓	✓		✓
*Stellera*	✓	✓	✓	✓
*Synaptolepis*		✓		
*Thymelaea*		✓		
*Wikstroemia*	✓	✓	✓	✓

## Isolation

The isolation of tigliane and daphnane diterpenoids from plants involves several steps. Firstly, the dried plant is extracted using an organic solvent, such as ethanol or methanol, followed by liquid–liquid partitioning. Since tigliane and daphnane diterpenoids have low polarity, they are partitioned into low-polarity solvents, including ethyl acetate, chloroform, petroleum ether, hexane, or dichloromethane [13, 19, 20]. Then, silica gel and ODS silica gel column chromatography are frequently employed to fractionate the extracts. Finally, isolation and purification are typically achieved by reversed-phase preparative HPLC, which uses acetonitrile/water or methanol/water mobile phases. During the process of isolating and purifying tigliane and daphnane diterpenoids, they are often contaminated by commonly occurring chemical constituents such as fatty acids, acylglycerols, and chlorophyll. In particular, when the chemical behavior of diterpenoids, fatty acids, and acylglycerols are similar, it is difficult to remove these impurities to isolate trace amounts of diterpenoids [21]. Based on our experience, the following methods can be employed as possible solutions during the isolation and purification process: (1) Ion exchange resin or basic resin-based column chromatography is useful to eliminate acidic fatty acids. (2) When partial separation can be achieved in HPLC, recycling HPLC is effective for purification. (3) Compounds that cannot be separated at all by reversed-phase HPLC are likely to be structural isomers. In such cases, purification by normal-phase HPLC using mobile phases such as *n*-hexane/ethyl acetate or chloroform/methanol mixtures should be considered [22].

**Fig. 7:**
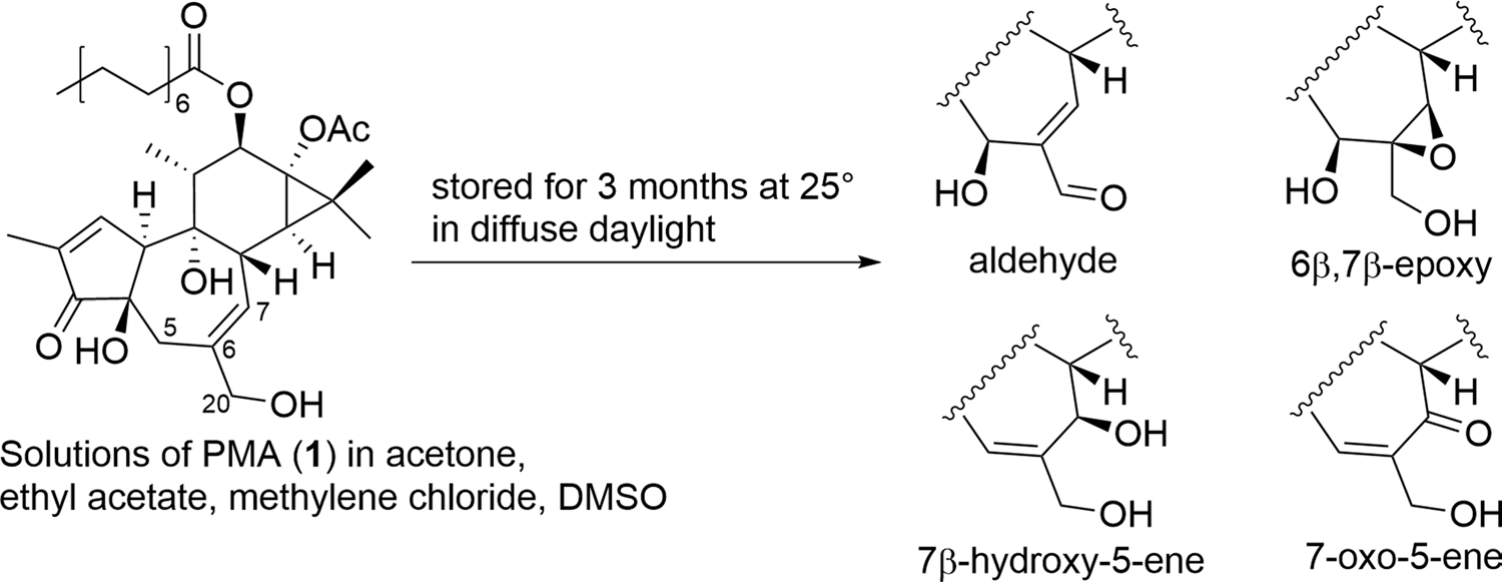
Autoxidation of tigliane diterpenoids

Regarding the stability of these diterpenoids during isolation, purification, and storage, it is desirable to store them in the dark under low-temperature conditions practically when kept in a solution. This is because autoxidation of the B-ring has been observed to occur in the solution state over a long period at room temperature for tiglianes (Fig. 7) [23]. On the other hand, in daphnanes, especially in compounds with a 3,5,20-trihydroxy structure, acyl transfer is likely to occur between these hydroxy groups. In polyhydroxy daphnanes, acyl transfer also occurs between the C-13 and C-14 hydroxy groups [24].

## Structure determination

The chemical structures of tigliane and daphnane diterpenoids have been primarily determined through NMR spectral analysis. Generally, these diterpenoids exhibit poor crystallinity, and there are limited reports of X-ray crystallographic analyses [25–28]. The ^1^H and ^13^C-NMR spectra of tigliane and daphnane diterpenoids share similarities but can be readily differentiated by observing the characteristic signals derived from the *gem*-dimethylcyclopropane group in tiglianes or the isopropenyl group in daphnanes, respectively.

### Tigliane diterpenoids

**Fig. 8:**
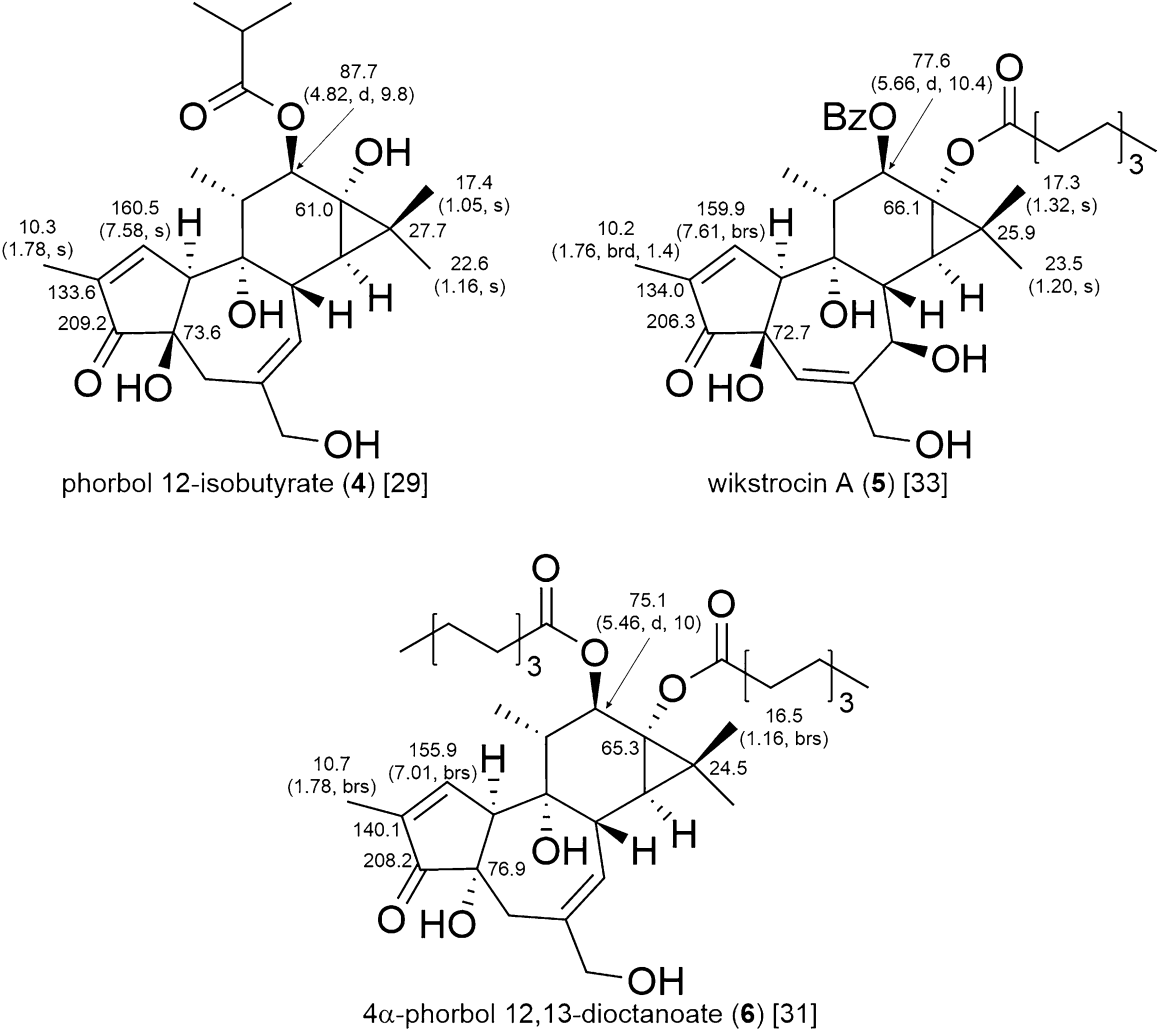
Diagnostic chemical shifts δ_C_ (δ_H_, multi, *J* in Hz) in CDCl_3_ of tigliane diterpenoids

In the ^1^H and ^13^C-NMR spectra of tiglianes, the characteristic resonances of the *gem*-dimethylcyclopropane moiety are observed as two singlet methyl proton resonances and an upfield shifted quaternary carbon resonance at ca. δ_C_ 22–28 [12]. All of the tiglianes isolated from plants of the Thymelaeaceae family possess an α,β-unsaturated ketone group in the A ring. Therefore, a downfield shifted resonance of olefinic proton and carbon resonances of the ketone group are observed. Moreover, methyl proton resonance of H_3_-19 is always observed as a broad singlet, broad doublet, or double doublet, since H_3_-19 is in long-range coupling with H-1 and H-10, when a double bond is located between C-1 and C-2. Generally, C-12 and C-13 have hydroxy or acyloxy groups attached to them, and the acyloxy group attached to C-12 can be easily determined by observing the HMBC correlation from H-12 to the esterified carbonyl carbon resonance. Since C-13 is an oxygenated tertiary carbon, it is difficult to determine the attachment of an acyloxy group by observation of the HMBC correlations. However, 13-hydroxytiglianes and 13-*O*-acylated tiglianes can be distinguished by observation of the chemical shift of C-13 of 13-hydroxytiglianes (**4**) at ca. δ_C_ 61, and of 13-*O*-acylated tiglianes (**5** and **6**) at ca. δ_C_ 63–67 (Fig. 8) [29]. The configurational analysis of tiglianes is generally performed by interpretation of NOESY (or ROESY) correlations and ECD Cotton effects, sometime combined with molecular modeling by quantum chemical calculations. The most common tiglianes have a *trans*-A/B/C ring structure, and observation of NOE (or ROE) from the α-oriented H-10 and β-oriented H-8 at the ring junction is useful for analysis of relative configurations. Although all of the tiglianes isolated from plants of the Thymelaeaceae family have a β-oriented 4-OH, a small number of tiglianes isolated from the Euphorbiaceae family have *cis*-fused A/B ring with α-oriented H-4/4-OH. The orientation of 4-OH can be determined by the chemical shift of C-4, at δ_C_ 76.9 for α-oriented 4-OH (**6**) and ca. δ_C_ 72–75 for β-oriented 4-OH (**4** and **5**) [30, 31].

ECD spectroscopy is commonly a useful tool to determine the absolute configuration of tiglianes. The Cotton effect of tigliane generally occurred in four regions: 320–340 nm (n → π* transition), 240–260 nm (*π* → π* transition), 220–240 nm (*π* → π* transition), and 200–220 nm (*π* → π* transition). The Cotton effect observed at 220–260 nm arising from the *π* → π* transition of the chromophore present in the A ring is useful in determining the absolute configuration [32]. Generally, in tiglianes with α,β-unsaturated ketone (C=C–C=O chromophore) and 4β-OH in the A ring, the Cotton effects are observed positive at 240–260 nm and negative at 220–240 nm. In addition, the differences in the B ring structure (differences in the position of hydroxy groups, ketone groups, or double bonds) may affect the Cotton effects at 220–260 nm and careful attention should be paid when analyzing the absolute configurations [33].

### Daphnane diterpenoids

**Fig. 9:**
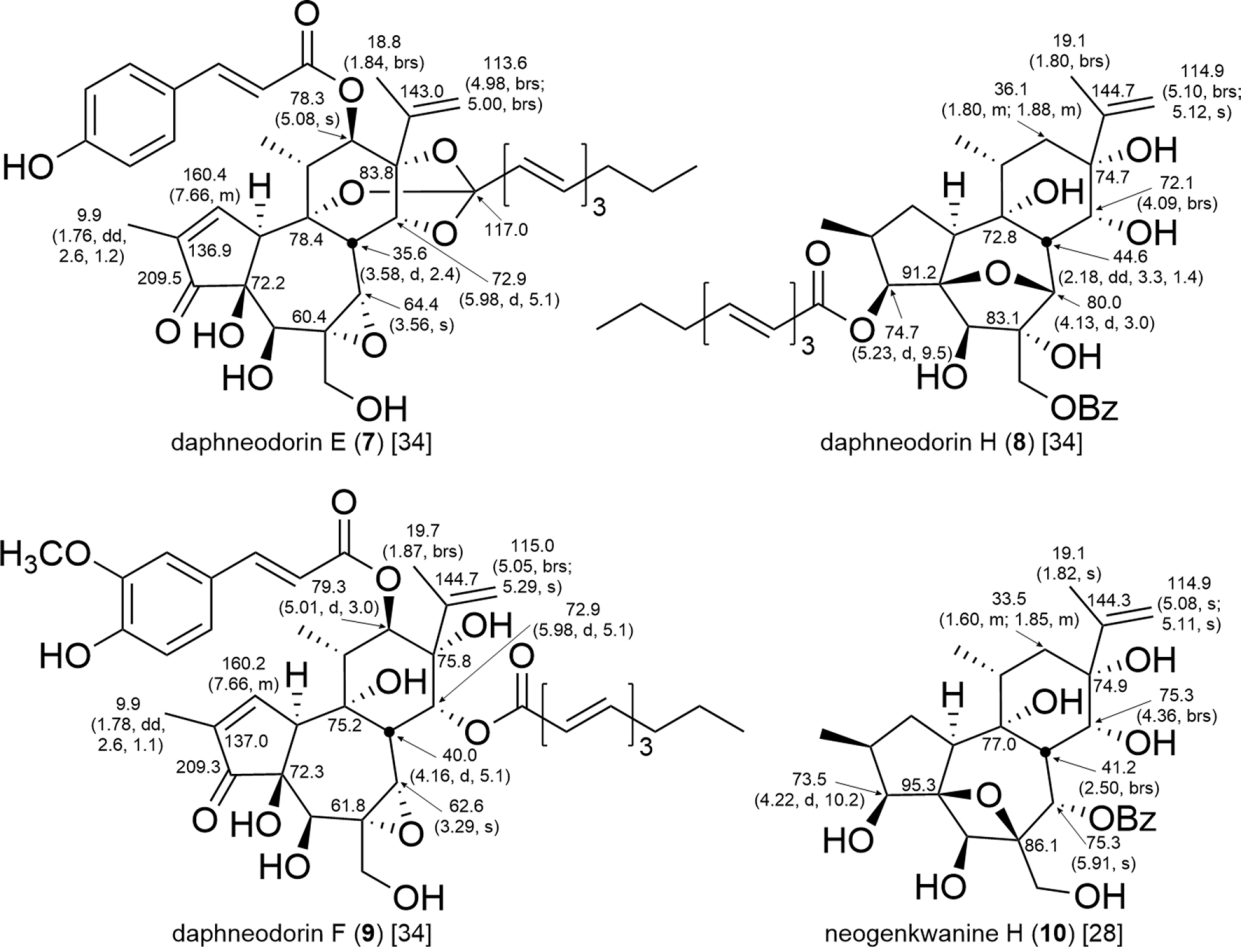
Diagnostic chemical shifts *δ*_C_ (*δ*_H_, multi, *J* in Hz) in CDCl_3_ of orthoester and polyhydroxy-type daphnane diterpenoids

In the ^1^H-NMR spectra of daphnanes, the characteristic resonances of the isopropenyl moiety are observed as an upfield shifted olefinic proton resonance of the terminal olefin and a methyl proton resonance in long-range coupling with the terminal olefin protons. Additionally, in orthoester daphnanes (**7**) and MDOs (**11** and **12**), a characteristic quaternary carbon resonance of the orthoester moiety is observed at ca. δ_C_ 116–120 in the ^13^C-NMR spectra [11]. Polyhydroxy daphnanes (**8**–**10**) are distinguished by the absence of the characteristic orthoester carbon resonances and the oxygenated carbon resonances at C-9, C-13, and C-14 downfield shifted compared to those in orthoester daphnanes (**7**) (Fig. 9) [28, 34].

**Fig. 10:**
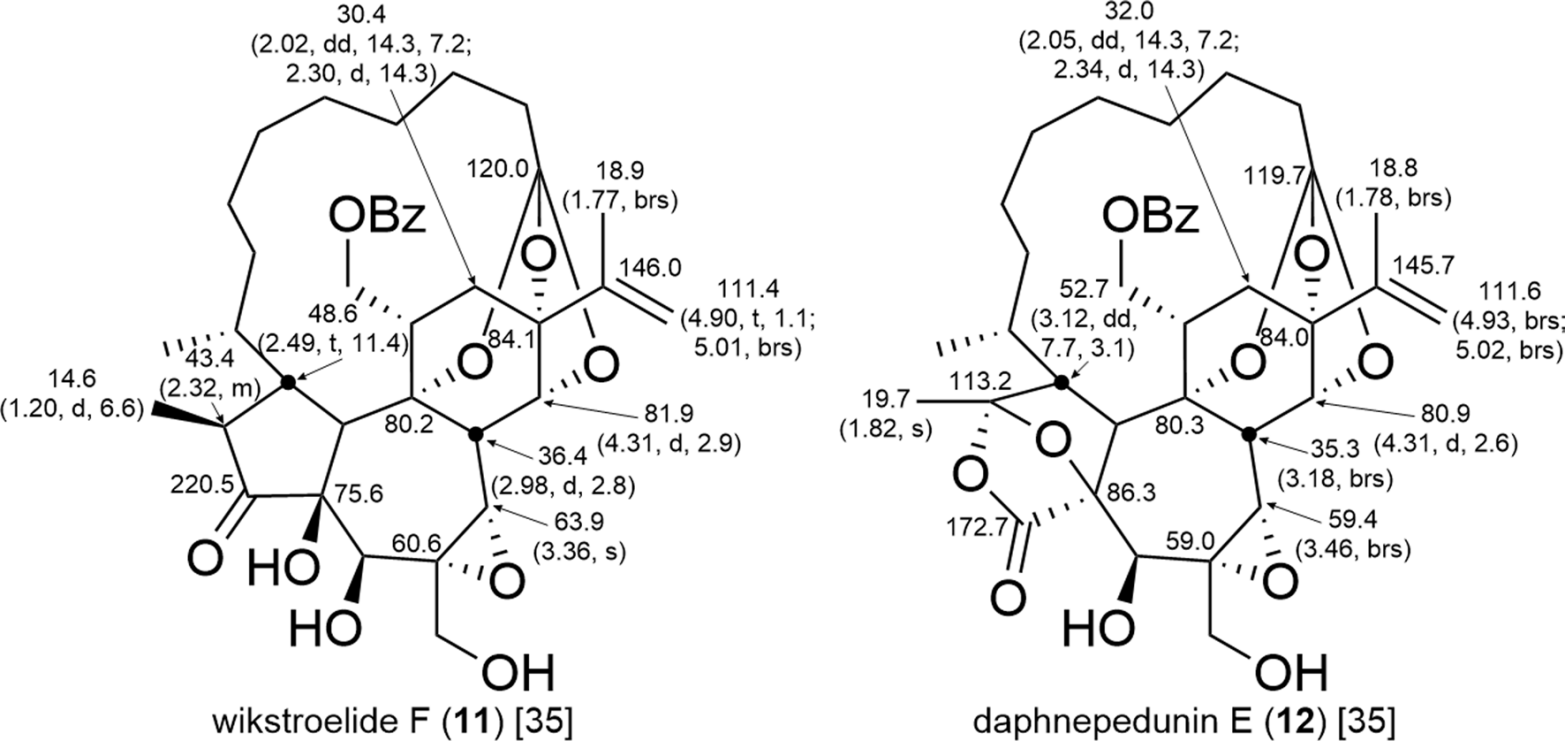
Diagnostic chemical shifts *δ*_C_ (*δ*_H_, multi, *J* in Hz) in CDCl_3_ of MDOs

The A-ring structure of orthoester daphnanes and polyhydroxy daphnanes resembles those of tiglianes, possessing a cyclopentenone or cyclopentanol structure. Consequently, the resonance of H-1 is observed as either an olefinic proton or a methylene proton resonance. On the other hand, in MDOs, the resonance of H-1 is observed as a methylene proton resonance due to carbon–carbon bond formation with the macrocyclic ring moiety at C-1. In addition to MDOs with a five-membered A-ring structure, some compounds (**12**) are with a bicyclo[2.2.1]heptane A-ring structure, in which the resonances of an acetal carbon at ca. δ_C_ 112–113 and a lactone carbonyl carbon at ca. δ_C_ 172–174 are observed in the ^13^C-NMR spectra (Fig. 10) [18, 19, 35–38]. In the B-ring, most daphnanes (**7**, **9**, **11**, and **12**) have an α-oriented 6,7-epoxy group, with the oxymethine carbon resonance of C-7 observed at ca. δ_C_ 59–65 ppm [39], and H-7 characteristically observed as a singlet, which may be attributed to a dihedral angle of approximately 90° between H-7 and H-8. The resonances of C-6 and C-7 in compounds with 6,7-dihydro, 4,6-epoxy, and 4,7-epoxy structure are downfield shifted from those with a 6,7-epoxy group. Furthermore, the resonance of C-4 is observed at ca. δ_C_ 90–92 for compounds (**8**) with a 4,7-epoxy structure and at δ_C_ 95.3 for the compound (**10**) with a 4,6-epoxy structure (Fig. 9). In daphnanes with an orthoester moiety, H-11α and H-12α in the C-ring are not coupled to each other since the dihedral angle between them is approximately 90°. Namely, in the case of a hydroxy or acyloxy group attached to C-12, H-12α is observed as a characteristic singlet. In the case of no substituent attached to C-12, H-12α is observed as a doublet due to coupling with H-12β, and H-12β is observed as a double doublet due to coupling with H-12α and H-11.

**Fig. 11:**
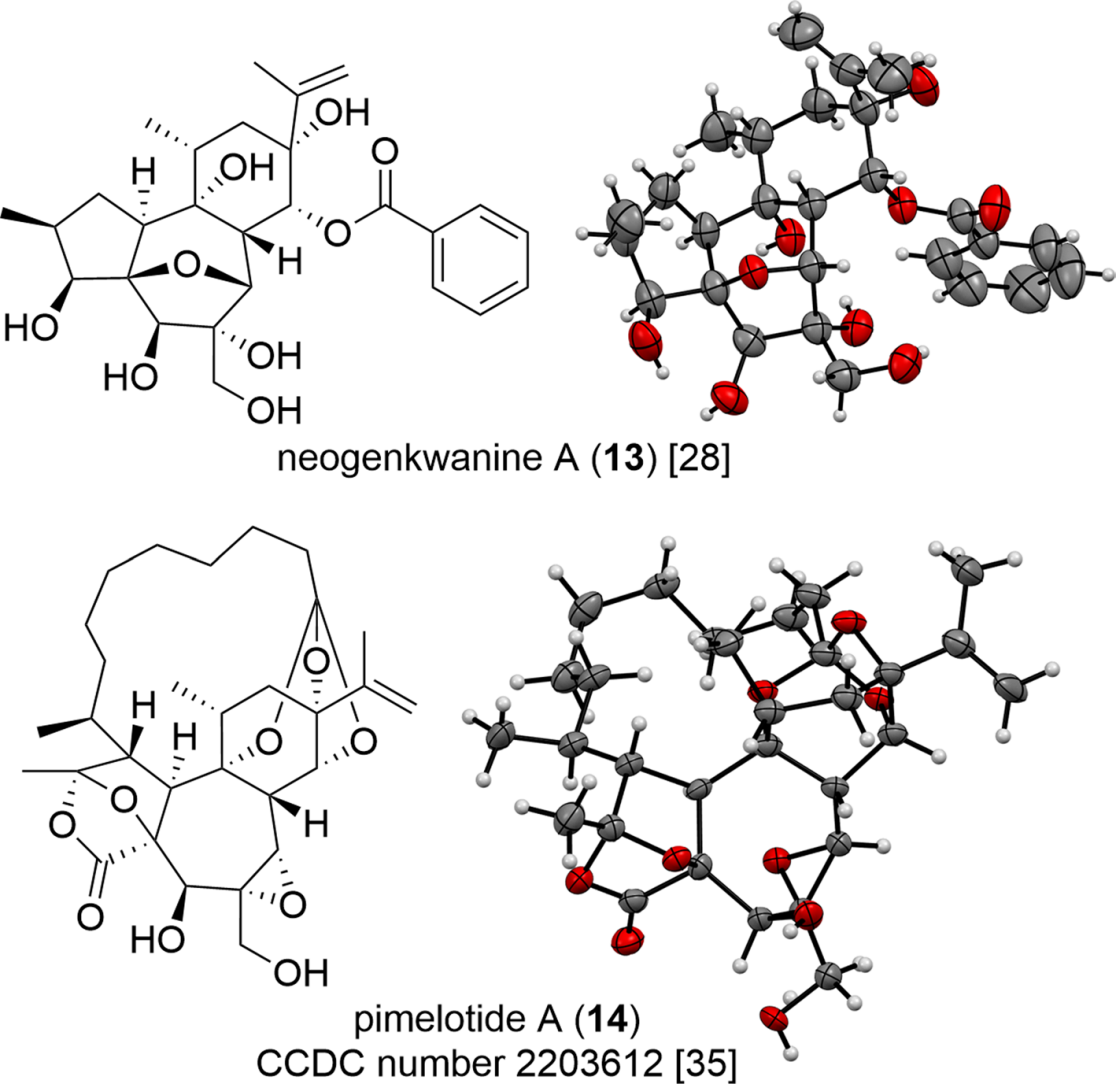
X-ray ORTEP drawing of neogenkwanine A (**13**) and pimelotide A (**14**)

Daphnanes have only been reported with *trans*-A/B/C ring structure so far, and their configurations are analyzed in the same way as that of tiglianes, but the ECD spectroscopy is not well applied for the analysis of the absolute configuration due to the lack of chromophore in the structure. While the number of daphnanes with characterized structures using X-ray crystallography is limited, there have been some compounds with diverse structural features that have been studied in this way. For example, polyhydroxy daphnane with a 4,7-epoxy structure (**13**) and MDO with a bicyclo[2.2.1]heptane A-ring structure (**14**) have been subjected to X-ray crystallography, providing valuable information about their absolute configuration and conformation (Fig. 11) [28, 35]. Determination of the relative configuration of alkyl groups branching on the macrocyclic ring part is a key point in the structural elucidation of MDOs, requiring careful analysis of NOE (or ROE). Recently, we reported a facile way to assign the relative configuration of the methyl group at C-9′ in the C_10_ aliphatic chain-type MDO by observing the chemical shift of C-10 or C-10′ [35].

### Structure analysis by ESI–MS/MS fragmentation

**Fig. 12:**
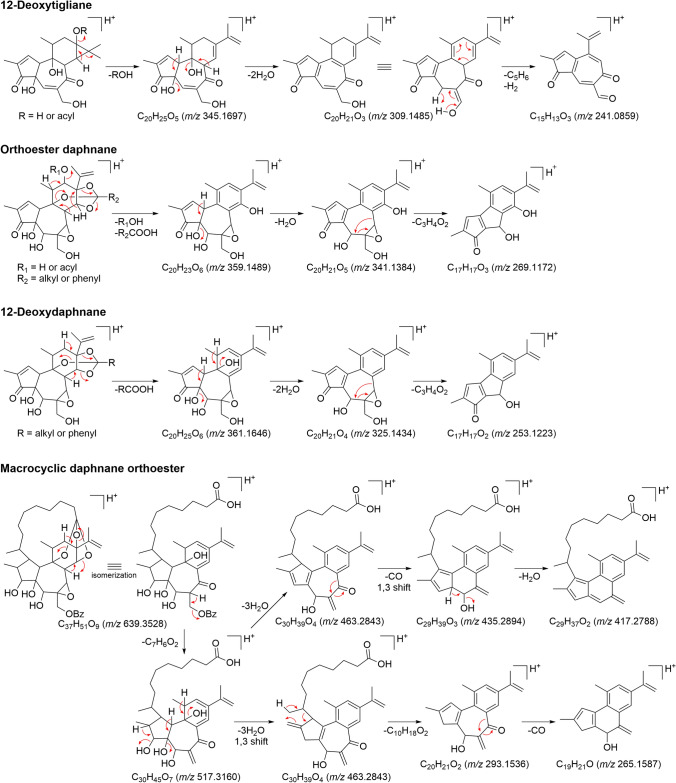
Proposed characteristic ESI–MS/MS fragmentation schemes of tigliane and daphnane diterpenoids

Recently, ESI–MS/MS fragmentation pathway analysis utilizing tandem mass spectrometry has been reported for tigliane and daphnane diterpenoids isolated from plants of the Thymelaeaceae family. Both tiglianes and daphnanes show abundant product ions in the positive mode, with a series of product ions observed at *m/z* 200–400 attribute to the consecutive losses of H_2_O and C=O from the C_20_ diterpene skeleton subsequent to the dissociation of the acyloxy and orthoester moieties (Fig. 12) [22, 40–42]. In tiglianes without a substituent attached to C-12, the characteristic product ion is observed in which the C_5_H_6_ unit is eliminated from the C-ring by the retro-Diels–Alder (RDA) reaction [42]. The characteristic product ion observed in daphnanes is the elimination of the C_3_H_4_O unit from the B-ring, which results in the fusion of a 7-membered ring to a 6-membered ring [40]. On the other hand, in the MDOs, the orthoester moiety is cleaved similarly to the orthoester daphnanes. However, abundant product ions are observed around *m/z* 400–500 due to consecutive losses of H_2_O and C=O while retaining the aliphatic chain in the A-ring [22]. As mentioned above, the ESI–MS/MS fragmentation of tiglianes and daphnanes is highly specific to their chemical structure. Therefore, LC–MS/MS analysis is a useful tool for the rapid identification of tiglianes and daphnanes in plants.

## Chemical synthesis

**Fig. 13:**
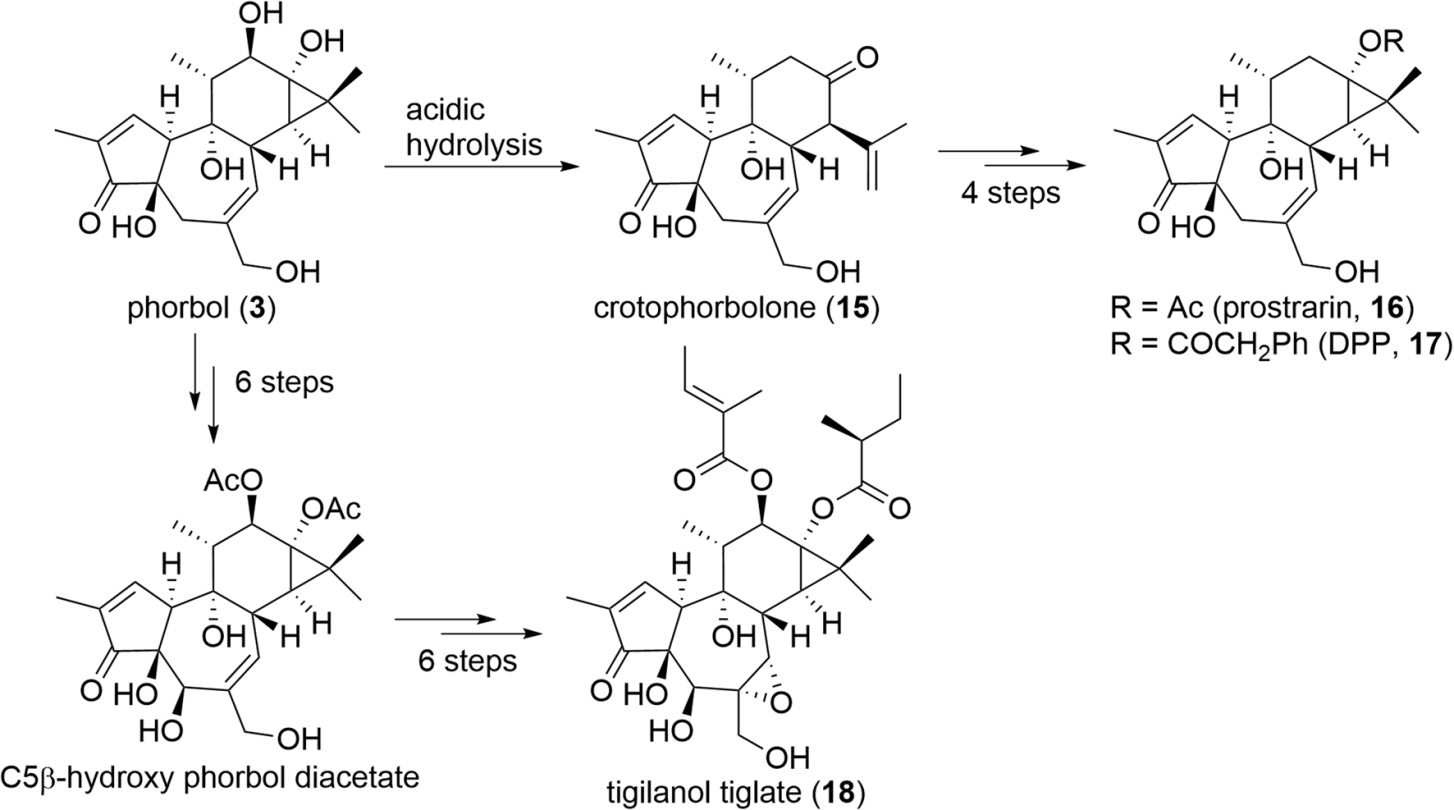
Wender’s practical semisynthesis of prostratin (**16**), DPP (**17**), and tigilanol tiglate (**18**)

**Fig. 14:**
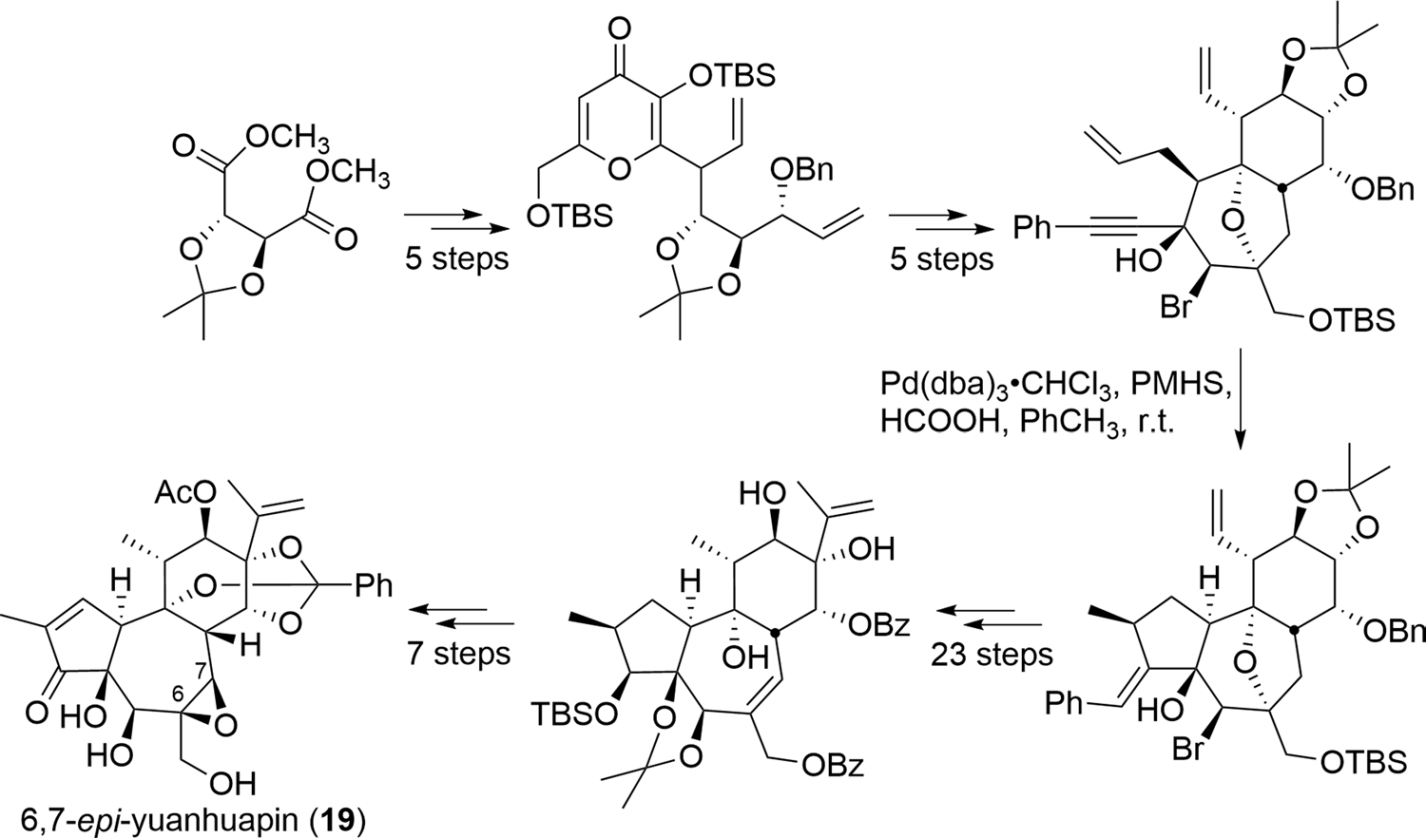
Wender’s total synthesis of 6,7-*epi*-yuanhuapin (**19**)

Tigliane and daphnane diterpenoids pose significant synthetic challenges due to their complex structures, but given their biological importance, numerous synthetic approaches have been reported by chemists [43]. The total synthesis of tigliane diterpenoids has been reported by several research groups, starting with the first total synthesis of phorbol (**3**) in 1989 [44, 45]. In 2008, a practical semi-synthetic method was developed to synthesize prostratin (**16**) and its derivative 12-deoxyphorbol-13-phenylacetate (DPP, **17**), which are drug candidates for the treatment of HIV infection. This method provides the synthesis of **16** and **17** in less than five steps from phorbol (**3**) or a rhamnofolane diterpenoid, crotophorbolone (**15**), which are readily available either naturally or synthetically (Fig. 13) [46]. This practical semi-synthetic method has made it possible to supply **16** and **17** on a gram basis, representing a significant step forward in drug development. More recently, tigilanol tiglate (EBC-46, **18**) with 5β-hydroxy-6α,7α-epoxy structure, which is a veterinary drug to treat dog cutaneous mast cell tumors, has been reported its synthesis in a high yield (12% overall yield) from phorbol (**3**) in only 12 steps. This method is also expected to be applied for synthesizing daphnanes with the same B-ring structure, which has not been achieved thus far [47]. In daphnane diterpenoids, since the first total synthesis of resiniferatoxin (**2**), an analgesic candidate with a 6-ene structure similar to phorbol (**3**), was reported in 1997, its synthetic route has been optimized and updated until 2022 [48–50]. The total synthesis of daphnanes with 6,7-epoxy structure has only been reported for 6,7-*epi*-yuanhuapin (**19**), but as mentioned above, the total synthesis of daphnanes with 5β-hydroxy-6α,7α-epoxy structure, which is primarily isolated from the plants of Thymelaeaceae family, has not yet been achieved (Fig. 14) [51]. Furthermore, the macrocyclic ring in MDOs increases the structural complexity, and despite attempts, linking the macrocyclic ring to the daphnane skeleton through chemical synthesis still remains a challenge [52].

## Biological activity

**Fig. 15:**
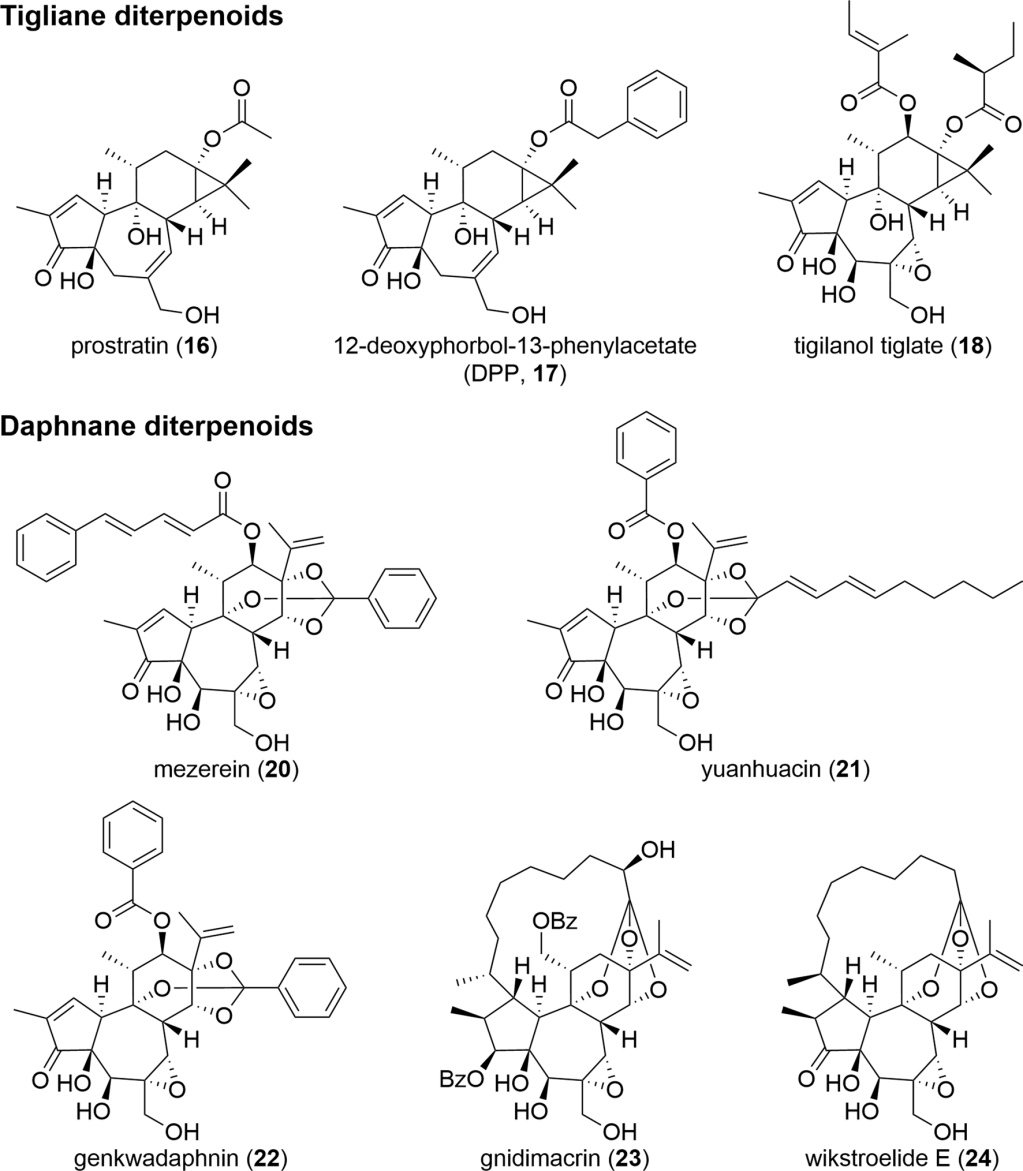
Representative tigliane and daphne diterpenoids with anti-cancer and anti-HIV activities

Tigliane and daphnane diterpenoids have been found to have a variety of attractive biological activities. Phorbol esters belonging to tiglianes are known to stimulate protein kinase C (PKC) by acting as a substitute for diacylglycerol, a naturally occurring second messenger. Daphnane diterpenoids with similar chemical structures are also considered to have the same function. PKC plays a role in the regulation of protein synthesis, DNA expression, and cell transformation [53]. The effects of tiglianes and daphnanes on PKC are known to include tumor-promoting and inflammatory activities, as described by PMA (**1**) [54]. However, these diterpenoids are not limited to “negative” biological activities. Some compounds (**16**–**24**) exhibit “positive” biological activities such as anti-cancer and anti-HIV without showing PKC-mediated tumor-promoting and inflammatory activities (Fig. 15). This difference in activity is thought to be due to the selectivity of these diterpenoids for the PKC isozymes [55].

### Anti-cancer activity

Since the PKC family plays a key role in cell proliferation and vasculature formation that is critical for tumor growth and has been identified as a direct target of “tumor-promoting” phorbol esters, inhibition of the PKC signaling pathway has long been a target of anti-cancer therapy [56]. However, recent studies have shown that PKC isozymes should be activated rather than inhibited in cancer therapy [57]. This finding emphasizes the importance of developing PKC activators for novel anti-cancer drugs [58–60]. Most recently, tigilanol tiglate (**18**), a tigliane diterpenoid, was approved by the US Food and Drug Administration (FDA) for the treatment of dogs with non-metastatic, cutaneous mast cell tumors [61]. Due to its efficacy, it is also being evaluated for human cancer treatment [62]. **18** was found to be highly selective for PKC isozymes, with particularly potent activation of PKCβ, weak activation of PKCα and PKCγ, and without activity against other isozymes. In vivo studies in mice demonstrated that the efficacy of **18** was inhibited by the PKC inhibitor bisindolylmaleimide 1 (BIM-1), suggesting that the anti-cancer activity of **18** is PKC-dependent [63].

**Fig. 16:**
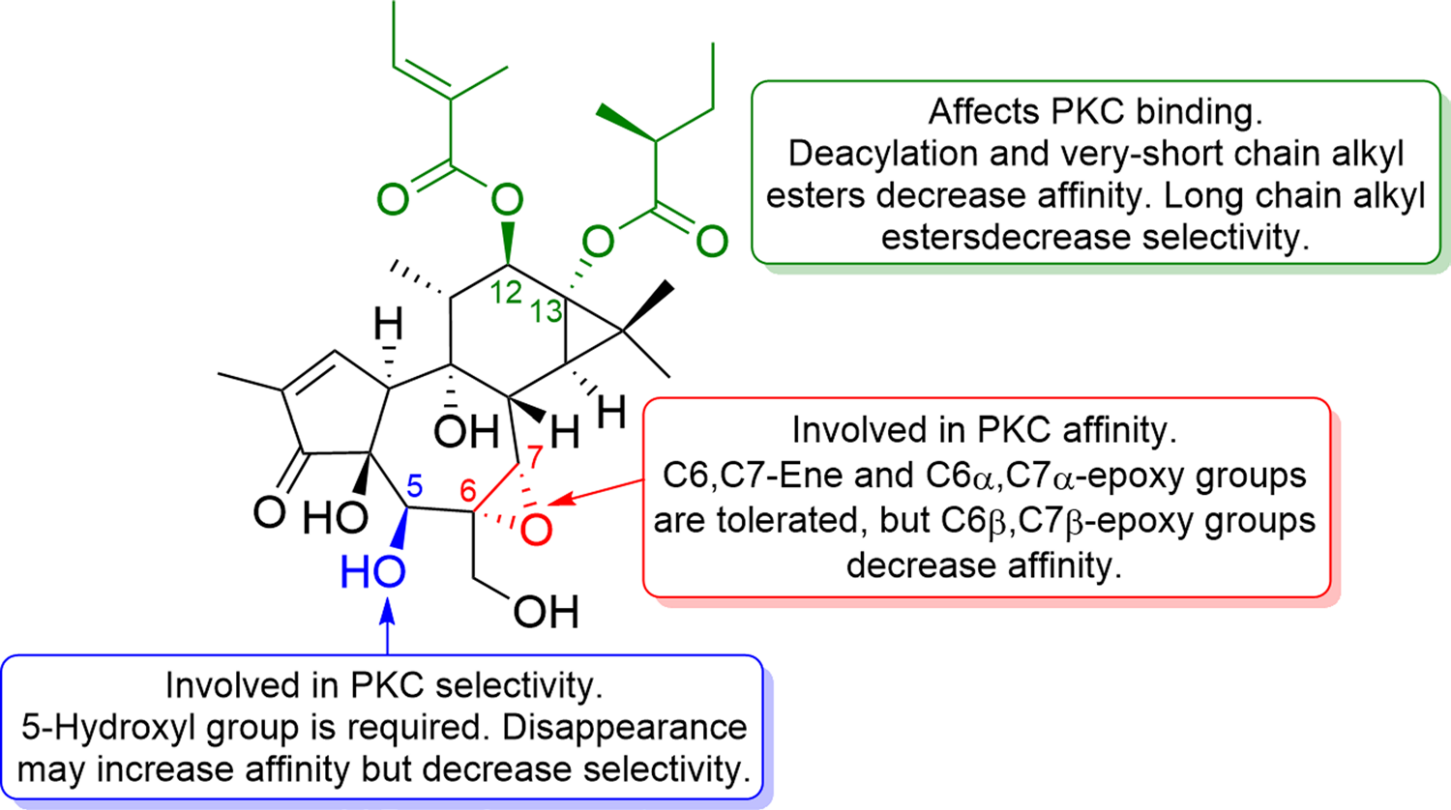
Structure–activity relationship correlations of tigilanol tiglate for PKC binding

**18** has a β-oriented 5-hydroxy group and an α-oriented 6,7-epoxy group in the B-ring. Initially, it was believed that the α-oriented 6,7-epoxy group was an important motif in the anti-cancer activity of **18** through selective activation of PKC [63]. However, recently the study of **18** revealed that both affinity and selectivity for PKCβ and PKCθ were maintained when an epoxy group is changed to a double bond, whereas when changed to a 6-ene structure, such as PMA (**1**), showed a significant increase in affinity for PKCβ and PKCθ, but the selectivity disappeared [47]. Although previous binding experiments between phorbol esters and the C1 domain of PKCδ have indicated that the oxygen functional groups at C-3, C-4, and C-20 are involved in binding to PKC, a series of validations using **16** and its derivatives revealed that the hydroxy group at C-5, as well as the α-oriented 6,7-epoxy group, are an important motif for PKC isozyme selectivity (Fig. 16) [64]. Furthermore, the different acyl groups attached at C-12 and C-13 affect the affinity for PKC and cytotoxicity against cancer cell lines, suggesting that the kind of ester substituent at the C-12 and C-13 is also important for binding to PKC and their anti-cancer activity [47, 65].

Since mezerein (**20**) was identified as a potent antileukemic compound from *Daphne mezereum* in the 1970s, the anti-cancer activity of daphnane diterpenoids attracted much attention [66]. Several daphnanaes, such as yuhuancin (**21**), genkwadaphnin (**22**), and gnidimacrin (**23**), have been reported to demonstrate favorable anti-cancer activity against various types of cancer cells in both in vitro and in vivo studies [67–80]. However, unlike tiglianes, no daphnanes has reached clinical application yet.

The antiproliferative activity of tigliane and daphnane diterpenoids on various cancer cell lines has been extensively studied in the literature [81–84]. However, evaluating their anti-cancer activity solely based on in vitro growth inhibition results requires caution. This is because the expression profiles of PKC isozymes in different cancer cell lines can vary significantly, and the IC_50_ values for the same compound can range from nM to µM depending on the evaluation protocol, particularly the duration of drug exposure [65, 85–87]. Additionally, studies have compared tigilanol tiglate (**18**) and PMA (**1**), revealing that the effects of this class of compounds on tumor cells in vivo may not be linked to their effects in vitro [63]. Several instances of variations in sensitivity to anti-cancer drugs in vitro and in vivo or in three-dimensional cell models have been documented, indicating that the in vivo efficacy of tigliane and daphnane diterpenoids might be mediated by different mechanisms than those observed in vitro. Thus, further careful investigation is necessary [88].

### Anti-HIV activity

Tigliane and daphnane diterpenoids exhibit another crucial biological activity through PKC activation, which is their anti-HIV activity. These diterpenoids display dual activity against HIV-1, inhibiting its replication, while also activating the latent HIV-1 in CD4^+^ T cells. This indicates their potential as latency-reversing agents (LRAs) which are of great interest for curing HIV-infection based on a “shock and kill” strategy [89]. The discovery of prostratin (**16**) has increased attention toward such activities.

Prostratin (**16**) was firstly isolated from *Pimelea prostrata* in 1976 and later rediscovered in *Homalanthus nutans* (commonly known as the Malala tree, Euphorbiaceae). It is a non-tumor promoter phorbol ester that exhibits potent cytoprotective activity against HIV-1-infected human lymphocyte cells [90–92]. Further studies have demonstrated that **16** not only reactivates latent infections but also inhibits HIV entry into host T cells by downregulating the expression of the HIV-1 receptor (CD4) and co-receptors (CXCR4 and CCR5) on the cell surface [93–95]. Structure–activity relationship studies have shown that altering the C-13 moiety of **16** and 12-deoxyphorbol-13-phenylacetate (DPP, **17**) affects latent HIV activation and affinity for PKC. The aromatic, relatively lipophilic unit is more favorable for anti-HIV activity [96–98]. Recent studies suggest that 12-benzoyloxyphorbol may be more effective than 12-deoxyphorbol in displaying anti-HIV activity [42]. However, current r studies on 12-benzoyloxyphorbols have been limited to in vitro HIV replication inhibition and HIV LTR-driven transcription activity, and further investigation into their latent HIV activation activity is needed [33, 42, 99–101].

In contrast to prostratin (**16**), the MDOs gnidimacrin (**23**) and wikstroelide E (**24**) exhibited about 1000 times greater potency in activating HIV-1 latent infection in cell lines like U1, ACH-2, and J-Lat [102, 103]. Mechanistic studies on **23** reveal its selective activation of PKCβ1 and PKCβ2, while not affecting PKCα, which is involved in tumor promoter activity, and PKCθ, which regulates T cell differentiation induction [104]. Furthermore, **23** did not induce T cell activation through CD3 and CD28, which leads to T cell depletion in HIV infection. It also does not trigger the production of inflammatory cytokines. E*x vivo* experiments conducted on peripheral blood mononuclear cells (PBMCs) from HIV patients with undetectable levels of HIV-1 after years of antiretroviral therapy (ART) reveal that even at concentrations as low as pM, **23** can significantly decrease HIV-1 DNA and the frequency of HIV-1-infected cells.

**Fig. 17:**
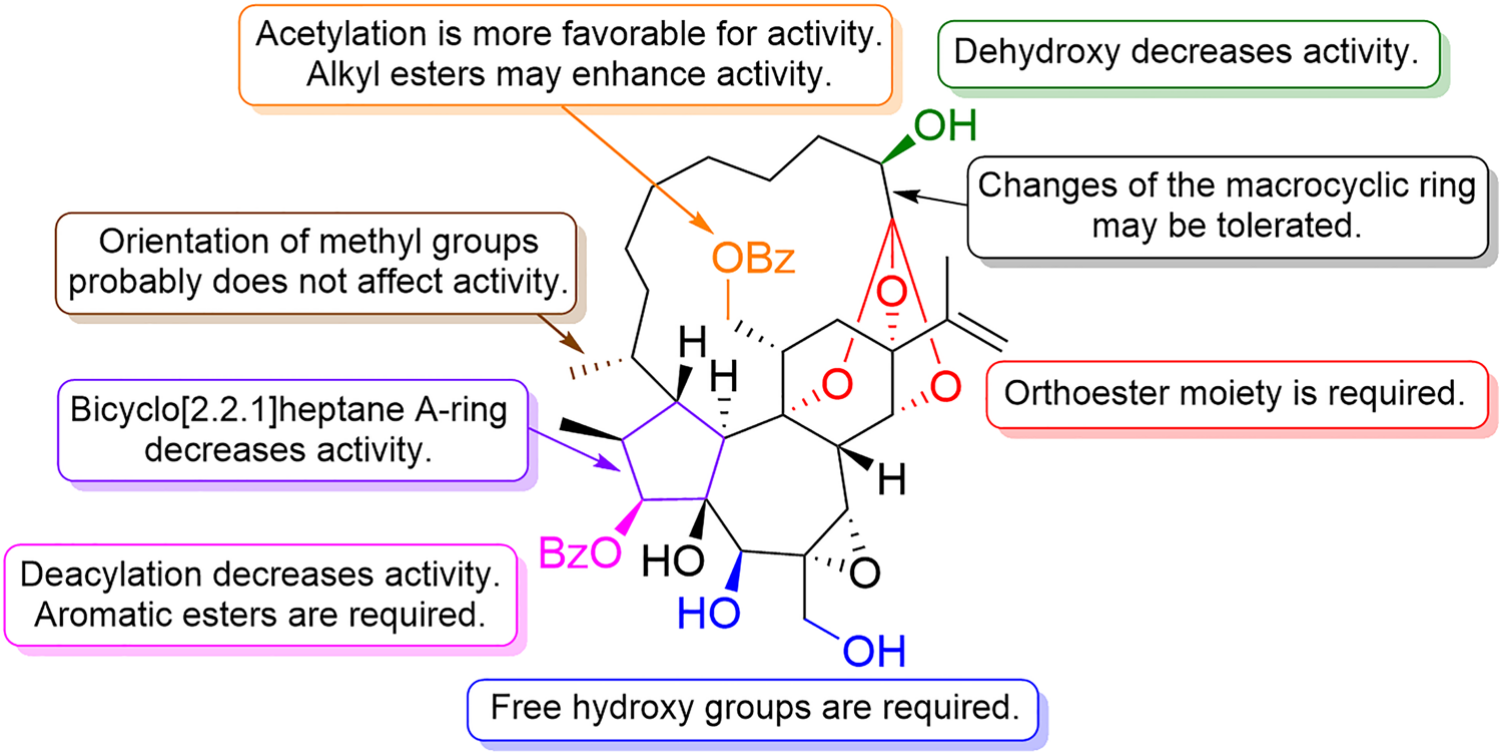
Structure–activity relationship correlations of daphnane diterpenoids for anti-HIV activity

The studies on **16** and **23**, which are tigliane and daphnane diterpenoids, to activate latent HIV are associated with their capacity to inhibit HIV replication. Studies on the structure–activity relationship of daphnanes for anti-HIV activity have revealed that the A-ring structure is crucial, with the five-membered A-ring structure being beneficial for anti-HIV activity, while compounds with a bicyclo A-ring structure decrease activity (Fig. 16) [35–37]. Among MDOs with a five-membered A-ring structure, compounds with a benzoyl group attached to the C-3 and hydroxy groups attached to the C-5, C-20, and C-2′ are important for the anti-HIV activity [101, 105]. In addition, orthoester daphnanes have potent HIV replication inhibitory activity, with an EC_50_ value of single digit nanomolar, which is more potent than the polyhydroxy daphnanes but less active than the MDOs [34, 36, 106]. These suggest that the macrocyclic ring part and orthoester moiety are essential motifs for enhancing the anti-HIV activity in daphnanes (Fig. 17).

## Conclusions

This review focuses on tigliane and daphnane diterpenoids isolated from plants of the Thymelaeaceae family, which have promising potential as anti-cancer and anti-HIV drug candidates. However, the complex chemical structures make their total synthesis a challenging task, and securing sufficient quantities of compounds remains a major obstacle. While some of these compounds have been/are in clinical trials, drug discovery research has not progressed well. Therefore, the development of efficient and practical total/semi-synthetic methods and further exploration of plant resources are eagerly awaited. Recently, target isolation using LC-tandem mass spectrometry and the molecular network has improved the efficiency of these diterpenoids [22, 42, 107–109]. It is hoped that further dereplication using LC–MS and molecular network strategies will lead to the discovery of new compounds with even more attractive chemical structures and biological activities. The establishment of a stable supply of these attractive diterpenoids is expected to lead to the development of novel pharmaceuticals.
